# A guanine-flipping and sequestration mechanism for G-quadruplex unwinding by RecQ helicases

**DOI:** 10.1038/s41467-018-06751-8

**Published:** 2018-10-10

**Authors:** Andrew F. Voter, Yupeng Qiu, Ramreddy Tippana, Sua Myong, James L. Keck

**Affiliations:** 1 Department of Biomolecular Chemistry, University of Wisconsin School of Medicine and Public Health, Madison, WI 53706 USA; 2 Department of Biophysics, Johns Hopkins University, Baltimore, MD 21218 USA

## Abstract

Homeostatic regulation of G-quadruplexes (G4s), four-stranded structures that can form in guanine-rich nucleic acids, requires G4 unwinding helicases. The mechanisms that mediate G4 unwinding remain unknown. We report the structure of a bacterial RecQ DNA helicase bound to resolved G4 DNA. Unexpectedly, a guanine base from the unwound G4 is sequestered within a guanine-specific binding pocket. Disruption of the pocket in RecQ blocks G4 unwinding, but not G4 binding or duplex DNA unwinding, indicating its essential role in structure-specific G4 resolution. A novel guanine-flipping and sequestration model that may be applicable to other G4-resolving helicases emerges from these studies.

## Abstract (web summary)

How G-quadruplexes (G4s) are resolved by helicases is still a matter of investigation. Here the authors provide mechanistic insight into G4s unwinding by presenting a crystal structure of resolved G4 DNA and the G4 binding domain of RecQ helicase from the bacterium *Cronobacter sakazakii*.

## Introduction

G-quadruplexes (G4s) are highly stable nucleic acid secondary structures that can form in guanine-rich DNA or RNA^1^. G-quartets, the repeating structures within G4s, are formed by an extensive hydrogen-bonding network that links four guanine bases around a cationic core. G4 structures, in turn, comprise G-quartets stacked upon one another, stabilized by base stacking between the layers. Their stability can make G4s impediments to numerous cellular processes, including replication^2^, transcription^3^, and translation^4^. Despite their potential hazards, G4-forming sequences are well represented in genomes, particularly within promoter regions^5^ and telomeric DNA ends^6,7^, indicating cells have developed mechanisms of abating the negative consequences of G4 DNA and have even co-opted the structures as regulatory and protective genomic elements.

G4 unwinding is essential for both G4 tolerance and G4 regulatory functions. Accordingly, cells have evolved a range of helicases that can unwind G4 structures, including DHX36^8^, the Pif1^2^ and XPD^9,10^ families of helicases, and members of the RecQ helicase family including bacterial RecQ^11^, yeast Sgs1^12^, and human WRN^13^ and BLM^14^. The importance of these helicases is highlighted by the profound genomic instability that results from their dysfunction, observed in xeroderma pigmentosa (XPD)^15^, Fanconi anemia (FANCJ (an XPD paralog))^16^, Werner (WRN)^17^ and Bloom (BLM)^18^ syndromes. In spite of the diverse clinical presentations caused by their absence, these enzymes operate on a range of G4 substrates using an apparent shared mechanism that relies on repetitive cycles of unwinding and refolding^19,20^. However, the small number of structural studies that have provided insights into the G4 unwinding process has limited our current understanding of the physical mechanisms underlying G4 resolution.

In this study, we report the X-ray crystal structure of the RecQ helicase from *Cronobacter sakazakii* (CsRecQ) bound to a resolved G4 DNA. Surprisingly, the 3′-most guanine base, which is the first base in the quadruplex that the 3′–5′ translocating RecQ would encounter, is bound in a guanine-specific pocket (GSP) in the helicase core. Residues within the GSP satisfy all of the hydrogen bonds that are normally formed by guanines within G-quartet structures, which highlights the remarkable guanine selectivity of the binding site. Guanine docking within the GSP is incompatible with a folded G4 structure, implying that the base must flip from the quartet to be sequestered within the GSP. Consistent with an important and selective role for the GSP in G4 unwinding, changes to the guanine-coordinating residues in RecQ block G4 DNA unwinding but do not alter duplex DNA unwinding. These data lead to a guanine-flipping and sequestration model of G4 unwinding by RecQ helicases that may also be shared with other G4 unwinding helicases.

## Results

### Structure of RecQ bound to a resolved G4

**Fig. 1:**
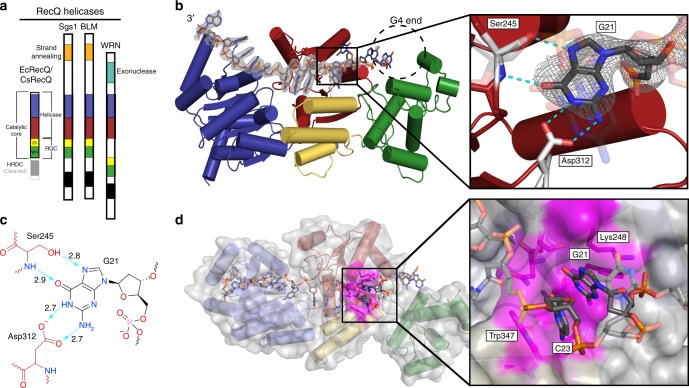
The guanine-specific pocket of the CsRecQ helicase. **a** Domain schematic representation of RecQ helicase family. RecQ comprises two RecA-like helicase folds (blue and red) and a C-terminal domain (RQC). The RQC contains a Zn^2+^-binding domain (Zn, yellow), a winged-helix domain (WH, green) and a helicase and RNaseD c-terminal domain (HRDC, gray). The HRDC has been removed in the RecQ catalytic core. **b** Crystal structure of CsRecQ bound to resolved G4 DNA. Domain colors correspond to **a**. *F*_o_ – *F*_c_ omit electron density contoured at 2.0*σ* is shown. The expected location of the G4 is highlighted. (Insert) The GSP in RecQ binds the flipped guanine with high specificity. Hydrogen bonds are represented by dashed lines. **c** Ligand interaction diagram of the GSP/guanine interface. Bond distances in Å are shown for the hydrogen bonds (teal). Residues from RecQ are red while the guanine is in black. **d** Surface representation of the CsRecQ bound to the resolved G4 with the GSP colored in magenta. (Insert) The flipped G21 is stabilized by hydrophobic interactions and base stacking with C23

To better understand how G4 structures are resolved by helicases, the catalytic core domain of CsRecQ (Fig. 1a) was crystallized in the presence of G4 DNA that included a 3′ single-stranded (ss) DNA loading site. An earlier structure of CsRecQ bound to duplex DNA with a 3′ ssDNA loading site showed that the enzyme’s helicase and winged-helix domains closed to form backbone interactions with the duplex, whereas the 3′ ssDNA end was bound in an electropositive channel in the helicase domain (Supplementary Fig. 1a)^21^. Because G4 and duplex DNA bind to the same surface of RecQ^22^, we hypothesized that RecQ would bind G4 DNA in the same orientation.

Surprisingly, the 2.2 Å-resolution structure revealed a product complex of CsRecQ bound to unwound G4 DNA rather than a folded quadruplex (Fig. 1b and Table 1). The RecQ/G4 product structure was very similar to the RecQ/duplex DNA structure, with a root mean square deviation of 0.68 Å among 511 Cα atoms (Supplementary Fig. 1). As was seen in the RecQ/duplex structure, the 3′ ssDNA is bound in an electropositive groove across the face of the helicase domain and it extends to dock in the ATP binding site of a symmetrically related molecule. Moreover, the helicase and winged-helix domains were closed around the unfolded G4. However, electron density was only observed for the three 3′-most guanines of the G4-forming DNA with the rest of the DNA apparently disordered within the crystal lattice. The positions of the resolved guanine bases deviated significantly from their expected placement within a folded G4, indicating that the quadruplex was unwound in the structure. The structure, therefore, suggested that binding by RecQ was sufficient to unwind G4 DNA, despite the presence of cations that otherwise stabilize the G4 (Supplementary Fig. 2).

**Table 1:** Data collection and refinement statistics

	RecQ-G4 (PDB 6CRM)
Data collection^a^	
Space group	P2_1_2_1_2
Cell dimensions	
* a*, *b*, *c* (Å)	78.5, 94.7, 98.9
*α*, *β*, *γ* (°)	90, 90, 90
Resolution (Å)	43.83-2.19 (2.27–2.19)^b^
* R*_sym_	0.15 (2.89)
* CC*_*1/2*_	0.999 (0.377)
* I* / *σI*	11.35 (0.76)
Completeness (%)	99.1 (92.63)
Redundancy	13.0 (11.3)
Refinement	
Resolution (Å)	43.83 – 2.19 (2.27–2.19)
No. of reflections	38101
* R*_work_ / *R*_free_	20.5/24.0
No. of atoms	
Protein	4035
DNA	290
Zn	1
Water	142
* B*-factors	
Macromolecules	87.55
Zn	54.49
Water	73.18
R.m.s. deviations	
Bond lengths (Å)	0.004
Bond angles (°)	0.67

^a^A single crystal was used for data collection

^b^Values in parentheses are for highest-resolution shell

### RecQ contains a guanine-specific pocket

Examination of the structure revealed an unexpectedly specific arrangement for binding to the unwound G4 product (Fig. 1b–d). The 3′-most guanine base of the G4-forming sequence (G21), which is the first base within the folded G4 that would be encountered by the 3′–5′ translocating RecQ enzyme, was found sequestered in a guanine-specific pocket (GSP) on the surface of RecQ. The GSP forms hydrogen bonds with the guanine base using the sidechain hydroxyl and backbone amide of Ser245 and the sidechain carboxyl group from Asp312 of RecQ (Fig. 1c). These contacts are uniquely selective for guanine and, strikingly, they substitute for all of the hydrogen bonds that stabilize guanines within G4 structures. The base is further stabilized by base stacking against a cytosine base two nucleotides 3′ of the flipped base (C23). The GSP is capped on the 5ʹ end by the hydrophobic portion of the Lys248 sidechain and by Trp347 on the 3′ side (Fig. 1d). Lys222 and Lys248 make additional contacts with the phosphodiester backbone of the unfolded DNA, anchoring it against the helicase domain (Supplementary Fig. 3). Given this arrangement, guanine-binding to RecQ is incompatible with its position within a folded G4. Instead it appears that the guanine must flip from within a G-quartet to be sequestered in the RecQ GSP. In both DNA-free and duplex DNA-bound bacterial RecQ structures, access to the GSP is occluded by Lys248, which folds to interact with Asp312 from the GSP^21,23^. However, the GSP is open to accept the guanine base in the RecQ/G4 product complex (Supplementary Fig. 4). These observations suggested a possible model in which guanine-flipping and GSP-mediated base-specific sequestration support RecQ unwinding of G4 DNA.

### Binding of RecQ variants to duplex and G4 DNA substrates

A guanine-flipping and sequestration model predicts that sequence changes in the GSP would impair G4, but not duplex, DNA unwinding. To test this prediction and allow for comparison with prior studies, *Escherichia coli* (Ec) RecQ (92.5% similar to CsRecQ, relevant residue numbering identical to CsRecQ) and CsRecQ catalytic core domain variants with compromised GSPs (Ser245Ala and Asp312Ala) were purified. The biochemical activity of these variants was tested alongside the wild-type EcRecQ and CsRecQ catalytic core domains. The CsRecQ Asp312Ala protein was unstable and difficult to purify, therefore, this protein was excluded from analysis.

Affinity for FAM-labeled duplex DNA with a 3ʹ ss extension was measured first for the RecQ panel (Supplementary Fig. 5a and Table 2). Each variant was found to bind the DNA, although the CsRecQ proteins had lower affinities relative to their EcRecQ counterparts. The EcRecQ Asp312Ala variant had a ~3–4-fold higher affinity for the partial duplex DNA, which may be due to the removal of a negative charge in the duplex DNA-binding groove. The DNA affinities reported here are consistent with those reported previously for the RecQ catalytic core^24^.

**Table 2:** DNA binding and unwinding rates of bacterial RecQ variants

RecQ variant	Duplex binding (*K*_d_, _app_, µM)	G4 binding (*K*_d_, _app_, µM)	Duplex unwinding rate (min^−1^)	G4 unwinding rate (TTA-T15) (min^−1^)	G4 unwinding rate (TAA-T15) (min^−1^)
EcRecQ	0.95 ± 0.06	1.0 ± 0.1	0.176 ± 0.017	0.038 ± 0.001	0.054 ± 0.002
Ec, Ser245Ala	2.2 ± 0.2	ND	0.19 ± 0.06	No unwinding	No unwinding
Ec, Asp312Ala	0.28 ± 0.02	0.33 ± 0.04	0.088 ± 0.005	No unwinding	No unwinding
CsRecQ	2.9 ± 0.3	4.7 ± 0.9	0.090 ± 0.013	0.14 ± 0.02	0.053 ± 0.002
Cs, Ser245Ala	4.0 ± 1.6	ND	0.081 ± 0.014	No unwinding	No unwinding

Values are reported as ± 1 standard deviation

*ND* not determined

Next, the affinity of each variant for G4 DNA with a 3ʹ ss extension was measured. EcRecQ, EcRecQ Asp312Ala, and CsRecQ all bound G4 DNA with very similar affinities to those measure with the partial duplex (Supplementary Fig 5b and Table 2). Unfortunately, we were unable to measure the equilibrium G4 affinity for either Ser245Ala variant; both were able to bind DNA but we observed a time-dependent decrease in anisotropy that made measurement of the binding constant impossible. This is likely due to a modest instability/insolubility of the variants in the conditions tested. Nevertheless, each of the variants could bind G4 and duplex DNA, indicating that residues within the GSP are not essential for G4 binding.

### Disruption of the GSP inhibits G4 but not duplex unwinding

**Fig. 2:**
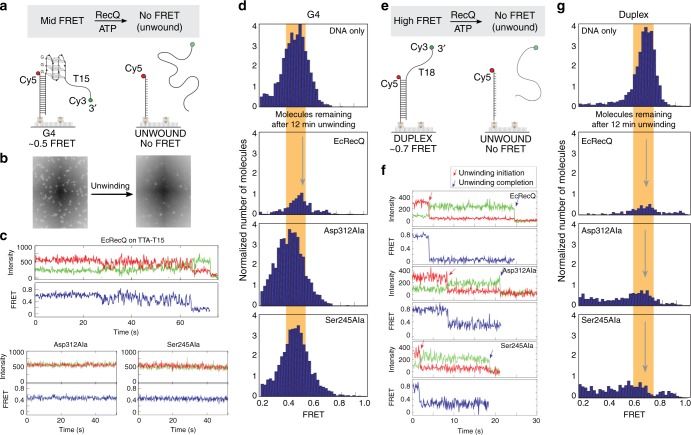
smFRET studies of RecQ helicase activity. **a** smFRET strategy to monitor RecQ-mediated unwinding of G4 DNA. **b** Representative field showing the loss of FRET signal following RecQ unwinding. **c** Representative smFRET traces of EcRecQ and the RecQ GSP variants on G4 DNA. The top traces for each RecQ variant represent the tethered Cy5 (red) and annealed Cy3 (green) signal, while the lower blue trace denotes the FRET efficiency. **d** Histograms of the smFRET signals for ~5000 G4 DNA molecules after a 12-min incubation with the specified RecQ variant. The orange bar and gray arrow denotes the primary FRET peak. **e** smFRET strategy to monitor RecQ-mediated unwinding of duplex DNA. **f** Representative smFRET traces of the action of EcRecQ and the RecQ GSP variants on the duplex DNA substrate. Traces are colored as in **c**. **g** Histograms of the smFRET signals for ~5000 duplex DNA molecules after a 12-min incubation with the specified RecQ variant. The orange bar denotes the primary FRET peak

Single-molecule (sm) FRET assays were carried out to determine the impact of GSP sequence changes on RecQ DNA unwinding. These assays were designed to test unwinding of substrates with a 3ʹ ss loading site that contain either a duplex structure preceded by a G4 element or a duplex structure alone (Fig 2a, e, respectively). The substrates consist of an immobilized Cy5-labeled 18mer annealed to a Cy3-labeled strand comprising the complementary 18mer along with either dT_15_ or both a G4 element and dT_15_. Unwinding of the substrate releases the Cy3-containing DNA strand and can be measured as a reduction of the number of Cy3 spots over time (Fig. 2b).

**Fig. 3:**
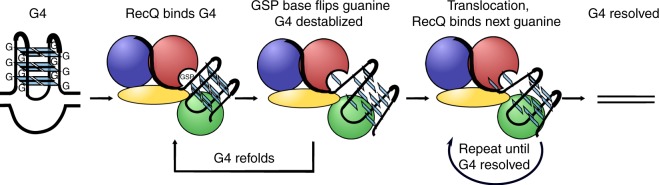
Model of RecQ-mediated G4 unwinding. RecQ (domains colored as in Fig. 1) binds the folded quadruplex, trapping it between the helicase and winged-helix domains. This positions the GSP near the G4, allowing for a guanine (indicated by blue squares) to be flipped out of the G-quartet and sequestered in the GSP. The guanine can either release back into the G-quartet, allowing the G4 to refold and leading to the observed repetitive FRET cycling, or RecQ can translocate to the next guanine

In this assay, both EcRecQ (Fig. 2c, d) and CsRecQ (Supplementary Fig. 6) were able to unwind substrates containing either of two antiparallel G4 DNAs, TTA-T_15_ (5′-TTA GGG TTA GGG TTA GGG TTA GGG-3ʹ) or TAA-T_15_ (5′-GGG TAA GGG TAA GGG TAA GGG-3′) (Table 2), using cycles of repetitive unwinding and refolding shown in the single-molecule trace (Fig. 2c, top, Supplementary Fig. 6). Repetitive unwinding/refolding cycles are marked by time-resolved high-amplitude FRET change signatures, such as that observed from ~30 to ~65 s with EcRecQ in Fig. 3c. Neither EcRecQ nor CsRecQ were active against cMyc, a parallel G4 DNA.

In contrast to the results with the wild-type RecQ proteins, none of the GSP variant RecQ proteins were able to unwind the G4 DNA structures. Single-molecule traces (Table 2, Fig. 2c, bottom, and Supplementary Fig. 6) showed that each of the GSP variants failed to elicit the repetitive unwinding/refolding FRET signature observed with the wild-type RecQ proteins and G4 unwinding was not observed, even after long (12 min) incubation periods. These data are consistent with an essential role of the RecQ GSP in G4 unwinding.

To test whether the GSP RecQ variants retained duplex helicase activity, the assay was repeated using a substrate that lacked the G4-forming sequence (Fig. 2e). The single-molecule traces (Fig. 2f and Supplementary Fig. 7) and FRET histograms before and after the addition of the proteins (Fig. 2g), demonstrate robust helicase activity of the duplex DNA substrate by all of the variants. Each protein unwinds the DNA at rates that were very similar to those observed with EcRecQ and CsRecQ (Table 2). Thus, the GSP in RecQ is required uniquely for unwinding G4 DNA.

In an attempt to visualize folded G4 DNA bound to RecQ, crystals of the Ser245Ala CsRecQ catalytic core variant were generated with G4 DNA. Diffraction data were collected from over a dozen crystals and molecular replacement revealed several crystals in which the guanine base was not found in the altered GSP. In these cases, discontinuous electron density consistent with the dimensions of a folded G4 structure was observed in the cleft formed by the helicase and winged-helix domains (Supplementary Fig. 8). Unfortunately, the fragmented nature of the electron density did not permit modeling of the full G4 structure. Nonetheless, the structural study was consistent with the significantly reduced activity of the variant predicted from the FRET experiment.

## Discussion

Despite the importance of G4 homeostasis in cells, our mechanistic understanding of quadruplex resolution has been hampered by a lack of structural information for G4-processing helicases. In this report, we have described the X-ray crystal structure of a RecQ helicase bound to a resolved G4. The structure identified a guanine-specific pocket, or GSP, in RecQ that sequesters a guanine base from the resolved G4. Guanine is selectively bound within the GSP via residues that form a pattern of hydrogen bond donors and acceptors that mimic the bonding pattern for a guanine within a G-quartet structure. As such, guanine-binding to the GSP is incompatible with a folded G4 structure and instead requires the base to be flipped away from the G4. These observations suggested a possible role for the GSP in G4 unwinding. In agreement with such a role, RecQ variants with altered guanine-binding residues failed to unwind G4 DNA, but they maintained their ability to unwind duplex DNA. Our data collectively support an unexpectedly specific helicase mechanism for RecQ unwinding of G4 structures that relies on guanine base flipping and sequestration for G4 resolution.

In the G4 unwinding model, RecQ first recognizes a ssDNA/G4 junction, placing the G4 in a position adjacent to the GSP and leaving the pocket poised to receive the 3′-most guanine from a G-quartet as it flips from the folded structure (Fig. 3). For the structural studies described here, guanine sequestration appears sufficient to unfold a G4 with three guanine quartet planes. ATP-dependent RecQ translocation would then slide the 3′-most guanine base out of the GSP, moving it along the face of the helicase domain and allowing the next guanine to be sequestered within the GSP as the G4 structure is resolved. What then gives rise to the repetitive cycles of G4 unwinding and refolding that have been observed in single-molecule experiments^11,14^? Two possibilities may explain this phenomenon. First, since RecQ must release the first guanine to advance along the DNA, it may be that the base can either slide along the ssDNA binding face of RecQ to promote unwinding or it can flip back and allow the G4 structure to refold (Fig. 3). It is possible that G4 reformation is more efficient than processive translocation, which would lead to repetitive rounds of unwinding and refolding. Second, although the GSP matches the hydrogen-bonding pattern for a guanine in a folded G4, it may form a complex that is less stable than that found in the context of a G4, which includes base stacking and ionic stabilization in addition to hydrogen bonding. If RecQ transiently captures a frayed guanine from the 3′ end of the G4 and if translocation is slower than the rate at which the guanine can transition back into the folded G4, this difference could allow the captured guanine to be released and the G4 to reform, resulting in a cycle of G4 unwinding and refolding.

Base-flipping activities have been observed in several enzymes that act on nucleic acids, including polymerases^25^, endonucleases^26^, glycosylases^27^, and methyltransferases^28^. In these enzymes, base flipping is accompanied by a distortion of B-form DNA near the flipped base, facilitating extraction of the base by the enzyme while extensive protein-DNA contacts hold the enzyme in position. Similarly to RecQ, base-flipping enzymes coordinate the isolated nucleobase through a hydrogen-bonding pattern that selects for the targeted base. This specificity allows repair enzymes, for example, to survey the integrity of the flipped base prior to initiation of a repair process. RecQ binding may similarly distort G4 DNA to allow guanine base flipping. It is also possible that RecQ simply traps transiently frayed guanine bases at the ssDNA/G4 interface. Additional studies are needed to examine these possibilities.

Because the RecQ GSP is specific for a canonical base, it is possible that the GSP may inadvertently sample guanines outside of G4 structures, hindering RecQ unwinding of guanine-rich duplex DNA. Indeed, RecQ pauses have been observed while unwinding GC-rich duplex DNA^29^, which could possibly result from guanine occupancy in the GSP. However, examination of the structure of the GSP reveals a mechanism that appears to counteract such non-productive base-flipping. In the absence of G4 DNA, Lys248 and Asp312 interact with one another to occlude access to the GSP (Supplementary Fig. S4). This closure is maintained when RecQ is bound to duplex DNA^21^. However, interaction with resolved G4 DNA appears to favor GSP opening through an interaction formed between Lys248 and the phosphodiester backbone of the G4 product. This interaction could make the GSP accessible to guanine bases under conditions where resolved or, presumably, folded G4 DNA is bound to RecQ. This interaction may attenuate guanine-binding by the GSP during duplex DNA unwinding while promoting it during G4 unwinding.

It remains to be seen how prevalent a guanine base-flipping mechanism is among G4 helicases. Among the bacterial RecQ helicases, the GSP sequence is conserved but not invariant. Some variability may be tolerated in the GSP while still allowing for G4 helicase activity. It may also be the case that the GSP is structurally conserved, even if the sequence homology is not invariant. For example, examination of the structure of BLM helicase, a human RecQ homolog with G4 helicase activity, reveals a potential GSP situated at the duplex/ssDNA junction comprising Ser965 and either Glu900 or Asp 997 (Supplementary Fig. 9a, b)^30^. We are unable to assess if the other RecQ-G4 helicases WRN or Sgs1 possess a GSP due to the lack of structures of their catalytic cores. However, even outside of the RecQ family, GSP-like pockets can be found. One instance is the bacterial helicase UvrD, which also contains a GSP-like structure poised to potentially receive a guanine flipped from a G4 substrate (Supplementary Fig. 9c)^31^.

While base-flipping described here provides a simple method of G4 resolution, other mechanisms may also exist. A very recent structure of G4 DNA in complex with the helicase DHX36 has been reported, suggesting a mechanism of G4 resolution in which the G4 is bound by the extended N-terminal DHX specific motif (DSM)^32^. This binding triggers repetitive conformational shifts in the G4 that are thought to reorganize and destabilize the quadruplex before ultimately releasing the resolved DNA in an ATP-dependent manner. The broader applicability of this mechanism may be limited to proteins with a DSM or analogous domain. Furthermore, the DSM best recognizes and unfolds parallel G4s, whereas this not a requirement of the GSP mechanism. Indeed, different RecQ helicases are known to unwind both parallel and antiparallel G4s^20,33^.

In summary, our studies have identified a remarkably specific mechanism for G4 DNA unwinding by RecQ DNA helicases. This model relies on base flipping in a manner that was first envisioned as a possible helicase mechanism shortly after the discovery of enzyme-mediated DNA base flipping^34^, although experimental evidence for such a mechanism has been lacking prior to the structural work described here. Discovery of this novel mechanism also underscores the apparent importance of G4 regulation by helicases in vivo. In what ways do the G4-specific functions of RecQ helicases impact cells? Several RecQ pathways have been linked to recognition and/or processing of G4 structures, including those involved in recombination regulation^35^ and telomere maintenance^36^ in eukaryotes, and antigenic variation in bacteria^37^. Investigations of the cellular activities of RecQ variants with selectively-blocked G4 resolution functions could pave the way to a better understanding of the general roles of G4 structures in vivo.

## Methods

### Protein purification

The catalytic core of CsRecQ and EcRecQ and all variants were overexpressed in Rosetta 2 (DE3) *E. coli* cells transformed with pLysS (Novagen, Darmstad, Germany) and a RecQ overexpression plasmid. Cells were grown at 37 °C in Luria Broth supplemented with 50 μg/mL kanamycin and 1 μg/mL chloramphenicol. Once the cells reached an OD_600_ of 0.6, protein expression was induced with 1 mM IPTG for 4 h at 37 °C before the cells were pelleted and stored at −80 °C. Cell pellets were resuspended in lysis buffer (20 mM Tris·HCl (pH 8.0), 500 mM NaCl, 1 mM 2-mercaptoethanol (BME), 1 mM phenylmethane sulfonyl fluoride, 100 mM dextrose, 10% (vol/vol) glycerol, 15 mM imidazole), lysed by sonication and clarified by centrifugation. The supernatant was incubated with Ni-NTA agarose resin at 4 °C before being washed extensively with lysis buffer. The N-terminally His-tagged proteins were eluted from the resin with elution buffer (lysis buffer containing 250 mM imidazole) before the His tag and HRDC domains were removed by overnight thrombin cleavage while the protein was dialyzed into dialysis buffer (20 mM Tris·HCl (pH 8.0), 300 mM NaCl, 1 mM BME, 10% (vol/vol) glycerol). The cleaved protein was diluted to 100 mM NaCl, loaded onto a HiPrep QFF ion exchange column (GE healthcare, Chicago, IL) and eluted with a 0.1–1 M NaCl gradient. RecQ-containing fractions were pooled, concentrated, and then further purified with an S-100 size exclusion column (GE healthcare) before dialysis into storage buffer (20 mM Tris·HCl (pH 8.0), 1 M NaCl, 4 mM BME, 40% (vol/vol) glycerol, 1 mM ethylenediaminetetraacetic acid) and stored at −20 °C.

### Structural studies

HPLC-purified DNA for crystallographic and RecQ-G4 binding studies (G4 DNA, Supplementary Table 1) was purchased from Integrated DNA Technologies (Coralville, IA, USA). Oligonucleotides were resuspended in 18 MΩ H_2_O and stored at –20 °C. CsRecQ catalytic core or the Ser245Ala variant at 6.5 mg/mL in minimal buffer [10 mM Tris·HCl (pH 8.0), 1 M ammonium acetate] was mixed with G4-forming sequence at a 1:1.2 protein:DNA ratio. The complex was combined at a 1:1 (vol/vol) ratio with mother liquor [70 mM sodium acetate·acetic acid (pH 4.9), 30% (vol/vol) glycerol, 10% (vol/vol) PEG 4000], and crystals were formed by hanging-drop vapor diffusion then flash-frozen in liquid nitrogen.

X-ray diffraction data were collected at the Advanced Photon Source (LS-CAT beamline 21ID-F) and were indexed and scaled using HKL2000^38^. The structure of the CsRecQ/G4 DNA complex was determined by molecular replacement using the CsRecQ/duplex DNA structure (PDB ID code 4TMU)^21^ as a search model in the program Phaser^39^ followed by rounds of manual fitting using Coot^40^ and refinement using PHENIX^41^. The quality of the electron density map of the refine structure is shown in Supplementary Fig. 10. Coordinate and structure factor files have been deposited in the Protein Data Bank (PDB ID code 6CRM [10.2210/pdb6CRM/pdb]). The Ser245Ala CsRecQ variant was phased by molecular replacement using the CsRecQ/G4 product complex as a search model in the program Phaser^39^ followed by rounds of manual fitting using Coot^40^ and refinement using Phenix^41^.

### DNA-binding assay

G4 DNA containing a 3′ FAM modification (F-G4) was solubilized to 50 µM in G4 folding buffer [10 mM Tris·HCl (pH 8.0), 100 mM KCl]. Using a heat block, the DNA was heated to 95 °C for 5 min, after which the block was removed from heat and allowed to cool to room temperature over approximately 4 h. Folded DNA was then stored at 4 °C. RecQ proteins were serially diluted from 20,000 to 0.6 nM in G4 binding buffer [20 mM Tris·HCl (pH 8.0), 100 mM NaCl, 1 mM MgCl_2_, 1 mM β-mercaptoethanol, 0.1 mg/mL bovine serum albumin, 4% (vol/vol) glycerol], then incubated with 5 nM F-G4 for 30 min at room temperature in a total volume of 100 µL. The fluorescence anisotropy of each sample was measured at 25 °C with a Beacon 2000 fluorescence polarization system. Measurements are reported in duplicate and error bars represent 1 SEM. Binding affinities and uncertainties were determined using Prism version 5.0c (GraphPad Software, La Jolla, CA, USA). Duplex binding assays were performed as the G4 binding assays using a 3ʹ FAM-labeled ssDNA (duplex 1) annealed to an unlabeled 18mer (duplex 2) to create a substrate with an 18-bp duplex with 3ʹ overhang of 12 nucleotides.^21^ Duplex binding assays were performed in triplicate and error bars represent 1 SEM.

### smFRET DNA substrates

ssDNAs with amino modifier at the labeling sites were purchased from Integrated DNA Technologies (Coralville, IA, USA). The DNAs were labeled using Cy3/Cy5 monofunctional NHS esters (GE Healthcare, Princeton, NJ, USA). Amino modified oligonucleotides (10 nmol in 50 mL ddH2O) and 100 nmol of Cy3/Cy5 NHS ester dissolved in dimethylsulfoxide were combined and incubated with rotation overnight at room temperature in the dark. The labeled oligonucleotides were purified by ethanol precipitation.

Both G4 and non-G4 substrates consist of 18 base pairs of dsDNA and a 3′ tailed ssDNA of specific sequence (Supplementary Table 1. For non-G4 DNA substrate, the 18mer DNA is immediately followed with a tail of dT_18_. For G4 DNA substrates, a G4 sequence is between the 18mer dsDNA and the dT tail. A Cy5-Cy3 FRET pair are placed at the junction and the 3′ end of the ssDNA, respectively.

DNA substrates were annealed by mixing the biotinylated and non-biotinylated oligonucleotides in a 1:2 molar ratio in T50 buffer [10 mM Tris·HCl (pH 8.0), 50 mM NaCl]. The final concentration of the mixture is 10 μM. The mixture was then incubated at 95 °C for 2 min followed by slow cooling to room temperature to complete the annealing reaction in just under 2 h. The annealed DNAs were stored at –20 °C and were diluted to 10 nM single-molecule stock concentration in K100 buffer [10 mM Tris·HCl (pH 8.0), 100 mM KCl] at the time of experiment.

### smFRET unwinding assays

A custom-built total internal reflect fluorescence microscope was used for the single-molecule unwinding assays. A solid state 532 nm laser (75 mW, Coherent CUBE) is used to excited the donor dye in the Cy3-Cy5 FRET pair used in FRET experiments. Emitted fluorescence signals collected by the microscope are separated by a dichroic mirror with a cutoff of 630 nm to split the Cy3 and Cy5 signals, which are then detect on an EMCCD camera (iXon DU-897ECS0-#BV; Andor Technology). Custom C + + programs control the camera and IDL software and are used to extract single-molecule traces from the recorded data. The traces are displayed and analyzed using Matlab and Origin software. All homemade codes are in the smFRET package available at the Center for the Physics of Living Cells (https://cplc.illinois.edu/software/, Biophysics Department, University of Illinois at Urbana-Champaign).

All unwinding experiments were performed in RecQ Reaction Buffer [20 mM Tris·HCl (pH 7.5), 50 mM KCl, 3 mM MgCl_2_, 1 mM ATP] with an oxygen scavenging system containing 0.8% vol/vol dextrose, 1 mg/mL glucose oxidase, 0.03 mg/mL catalase1, and 10 mM Trolox. All chemicals were purchased from Sigma Aldrich (St. Louis, MO).

Biotinylated FRET DNA (50 to 100 pM) were immobilized on polyethylene glycol-coated quartz surface via biotin-neutravidin linkage. RecQ and mutant proteins (100 nM) were added at room temperature to initiate unwinding. 10–20 short movies (10 s) and 3–4 long movies (3 min) were then taken monitoring the Cy3 and Cy5 emission intensities over time. These are then analyzed to produce the FRET histograms and trajectories to monitor any unwinding activity.

To calculate the unwinding rate, note that as the DNA is unwound, the Cy3 strand is freed from the immobilized DNA substrate and the Cy3 signal disappears. Snapshots of the Cy3 spots detected in an imaging area are taken via short movies (2 s) and the spots counted over time. The counts are then plotted and fitted to an exponential curve to obtain the rate of disappearance of the Cy3 spots over time as the indication of unwinding. For each rate calculation, 400–500 single molecules were monitored and the standard error of the measurement was reported. During imaging, a fraction of the G4 molecules were unwound by a protein-dependent and GSP-independent mechanism. The number of G4s lost during through this process (~20% over 12 min) was insufficient to allow for rate calculations and the GSP-independent unwinding was assumed to be negligible relative to the GSP-dependent mechanism.

### Circular dichroism

G4 DNA used for the crystallographic studies were refolded by diluting to 10 μM in either 10 mM Tris·HCl (pH 8.0) or 35 mM sodium acetate-acetic acid, 500 mM ammonium acetate, 4% (vol/vol) PEG 4 K and 15% (vol/vol) glycerol by heating to 95 °C for 10 min and slowly cooling to room temperature. These conditions represent unfolded ssDNA or crystallization conditions, respectively. CD spectra were recorded on an AVIV 420 circular dichroism spectrometer at 25 °C over a range of 200–340 nm in a 1-mm path length quartz cuvette. Data were collected using a 1 nm step size with a 5 s average and a blank reading containing no DNA was subtracted from each reading.

## Electronic supplementary material

Supplementary Information

## Data Availability

The RecQ/G4 product structure is available at the Protein Data Bank, PDB ID: 6CRM [10.2210/pdb5XRN/pdb]. Other data are available from the corresponding author upon reasonable request.
