# Supplementary material for: A guanine-flipping and sequestration mechanism for G-quadruplex unwinding by RecQ helicases
Source: Nat Commun. 2018 Oct 10;9:4201. doi: 10.1038/s41467-018-06751-8 (PMC6180126; doi:10.1038/s41467-018-06751-8)
Supplement: Supplementary file 1 — Supplementary Information [file 41467_2018_6751_MOESM1_ESM.pdf]

Supplementary Information for

A guanine-flipping and sequestration mechanism for G-quadruplex unwinding by RecQ helicases

Authors: Andrew F. Voter[1], Yupeng Qiu[2], Ramreddy Tippana[2], Sua Myong[2], James L. Keck[1]

Supplementary Figure 1.

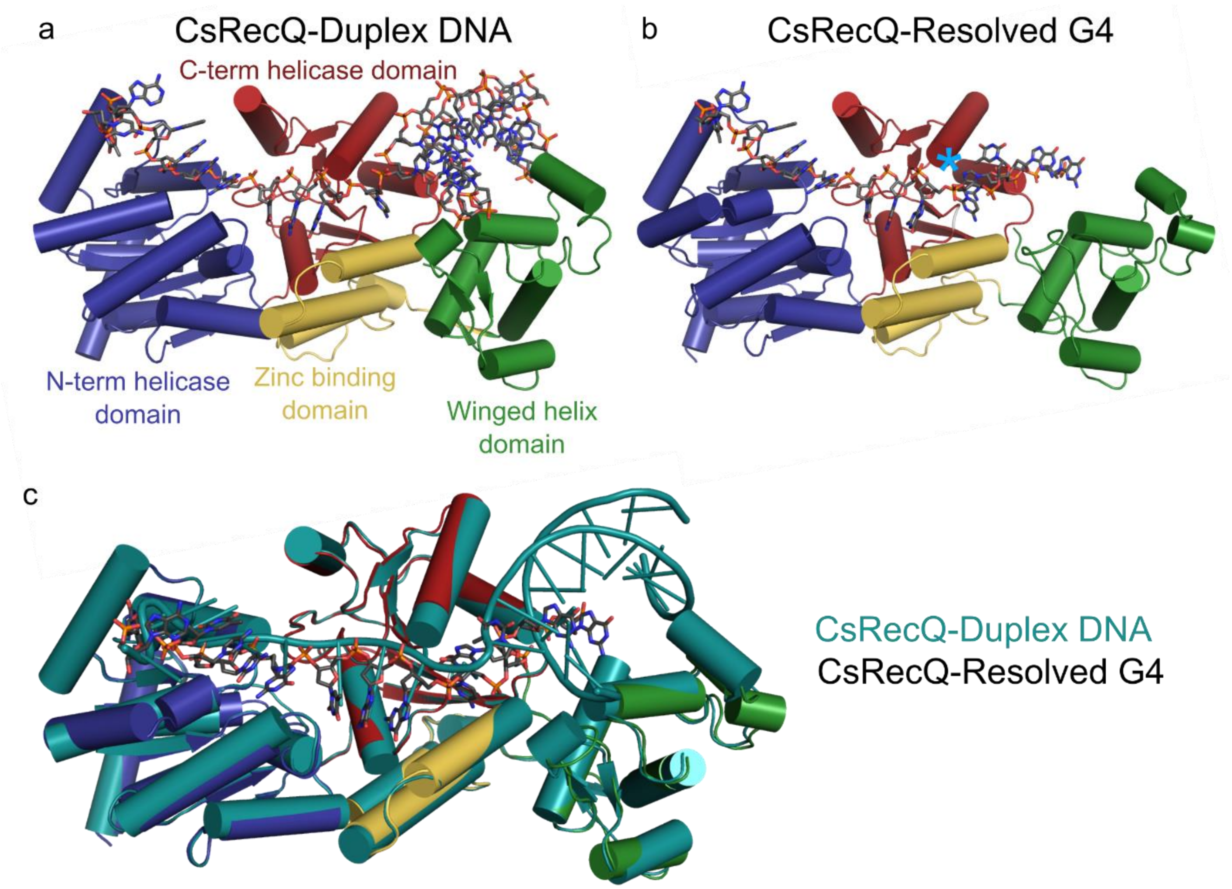

Supplementary Figure 1. RecQ adopts a similar confirmation when bound to duplex DNA or a resolved G4. a) Structure of CsRecQ bound to duplex DNA (PDB 4TMU)[1]. b) Structure of CsRecQ bound to a resolved G4 (PDB 6CRM). The flipped guanine is marked by a teal asterisk c) Overlay of the structure from a and b. The CsRecQ-Duplex DNA is colored in teal with the CsRecQ/G4 product structure colored as in b.

Supplementary Figure 2.

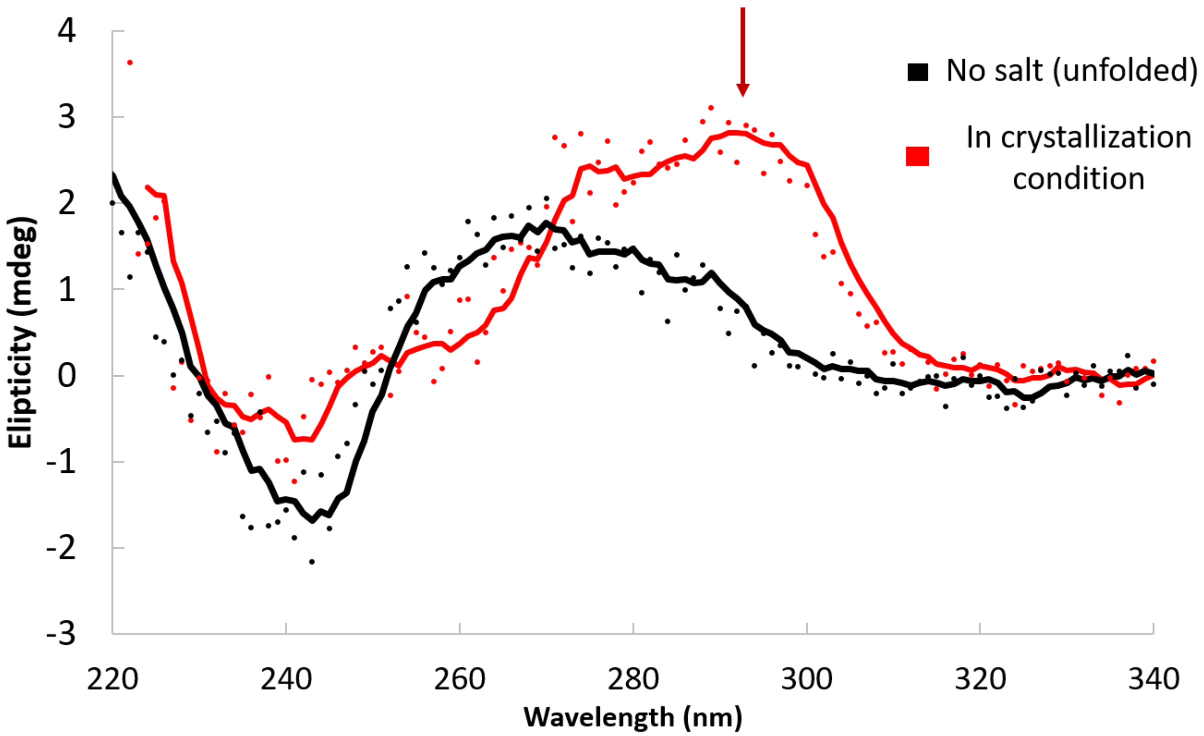

Supplementary Figure 2. Crystallization conditions support antiparallel G4 formation. Circular dichroic spectra of G4-folding DNA in either no salt conditions (black) or the crystallization conditions (red). Line represent the rolling average of the CD values shown by the individual points. Presence of a positive CD signal at 290 nm (arrow) indicates the formation of antiparallel G4 DNA.

Supplementary Figure 3.

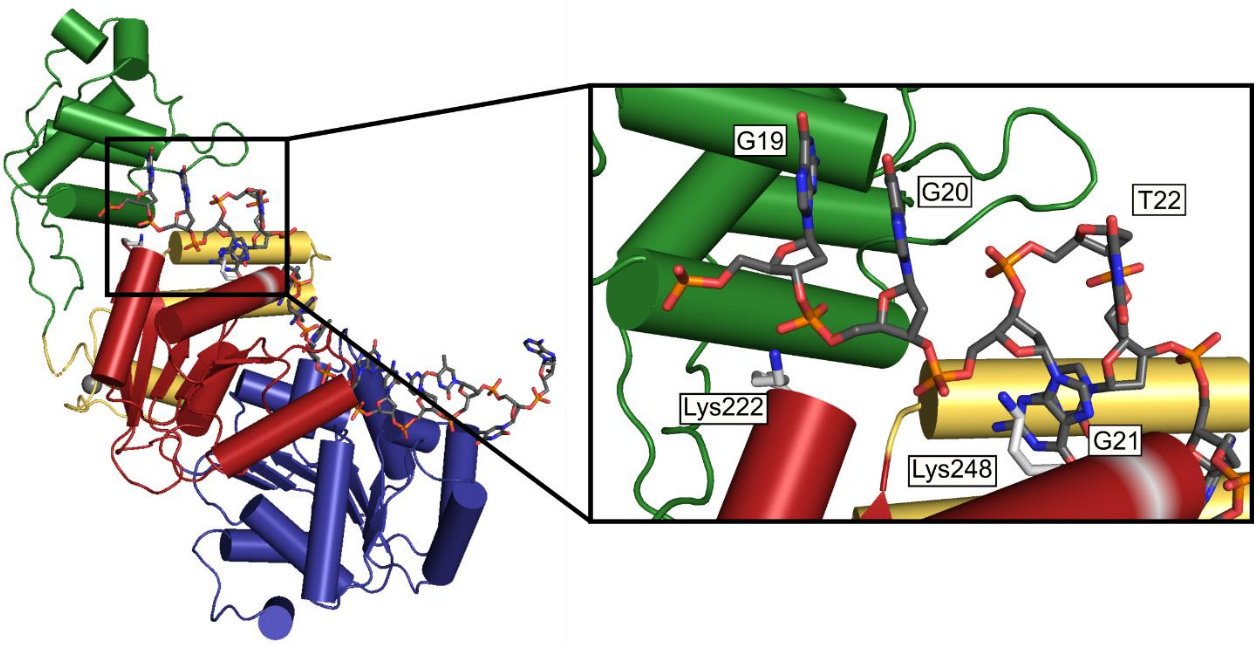

Supplementary Figure 3. Interaction between RecQ and the resolved G4. The resolved G4 DNA (gray sticks) is held against the face of the CsRecQ helicase domain (red cartoon) by Lys222 and Lys248 (white sticks).

Supplementary Figure 4

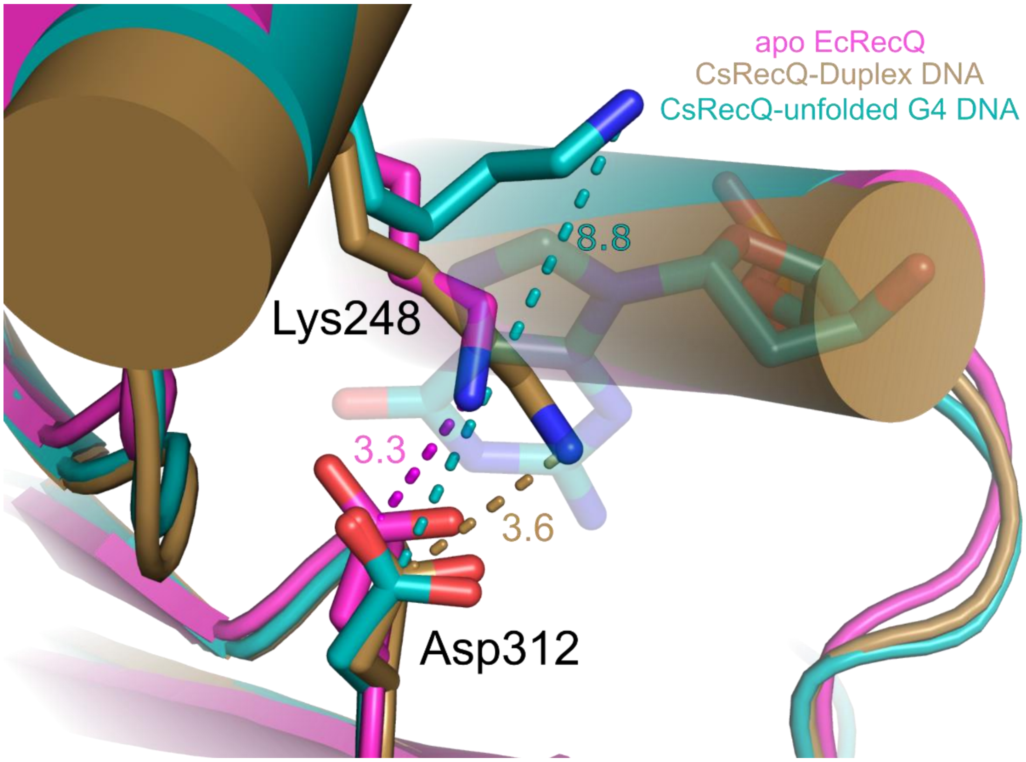

Supplementary Figure 4. The RecQ GSP is closed unless bound to resolved G4 DNA. The GSP in both apo EcRecQ (magenta, PDB 1OYW[2]) and duplex DNA-bound CsRecQ (yellow, PDB 4TMU[1]) is closed by interaction between Lys248 and Asp312, but is opened in the G4 product complex (teal, PDB 6CRM). Dashed lines and distances (Å) denote the distances between the ε-amino group of Lys248 and the carbon of the carboxylic acid group of Asp312.

Supplementary Figure 5.

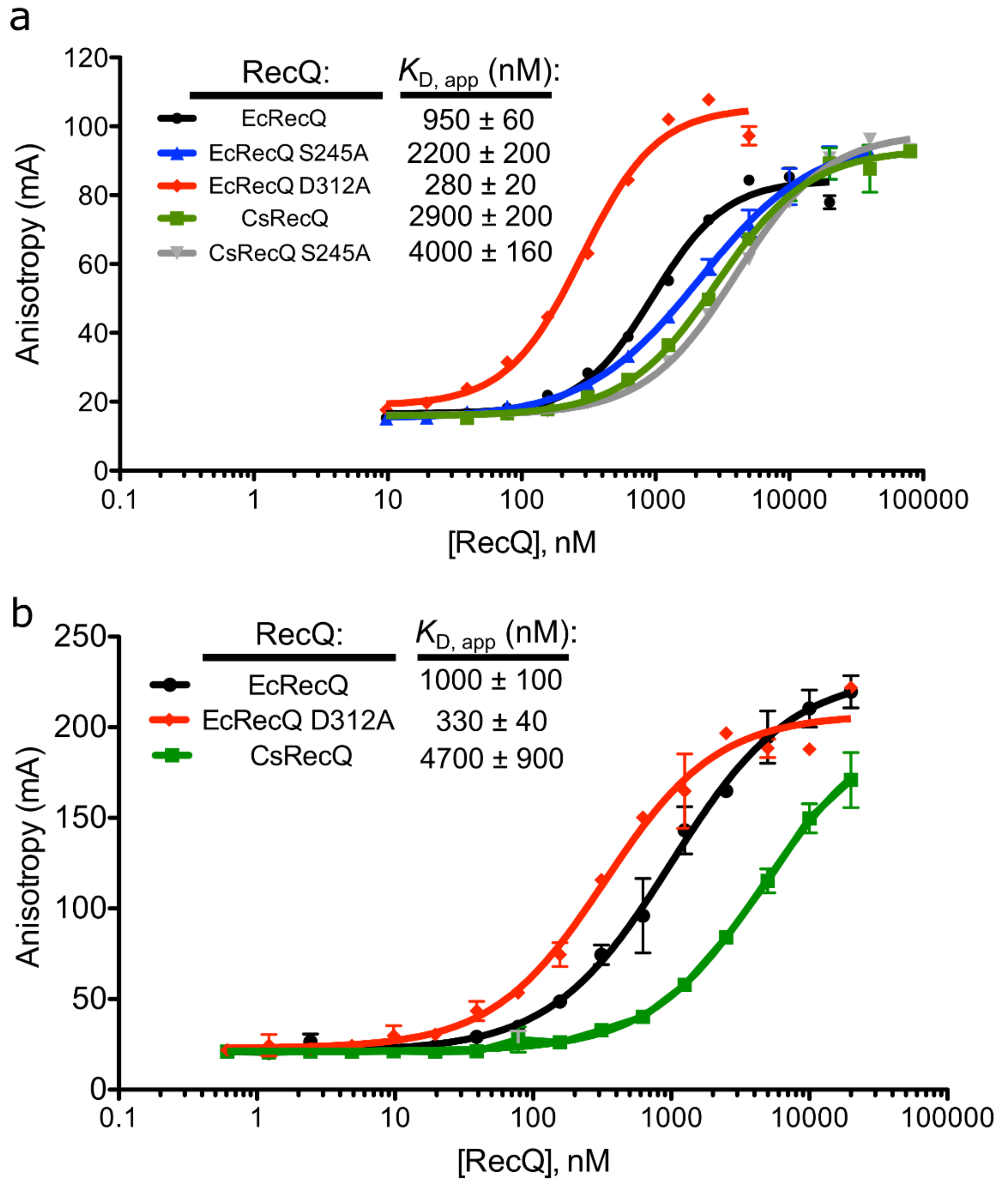

Supplementary Figure 5. Duplex and G4 binding of RecQ variants. a) Fluorescence anisotropy of folded G4 DNA incubated with increasing concentrations of each RecQ variant. Apparent dissociation constants ($K_{D, app}$) are reported ± 1 standard deviation. Error bars represent the SEM of 2 replicates. b) Fluorescence anisotropy of duplex DNA substrates incubated with increase concentrations of each RecQ variant. Error bars represent the SEM of 3 replicates.

Supplementary Figure 6.

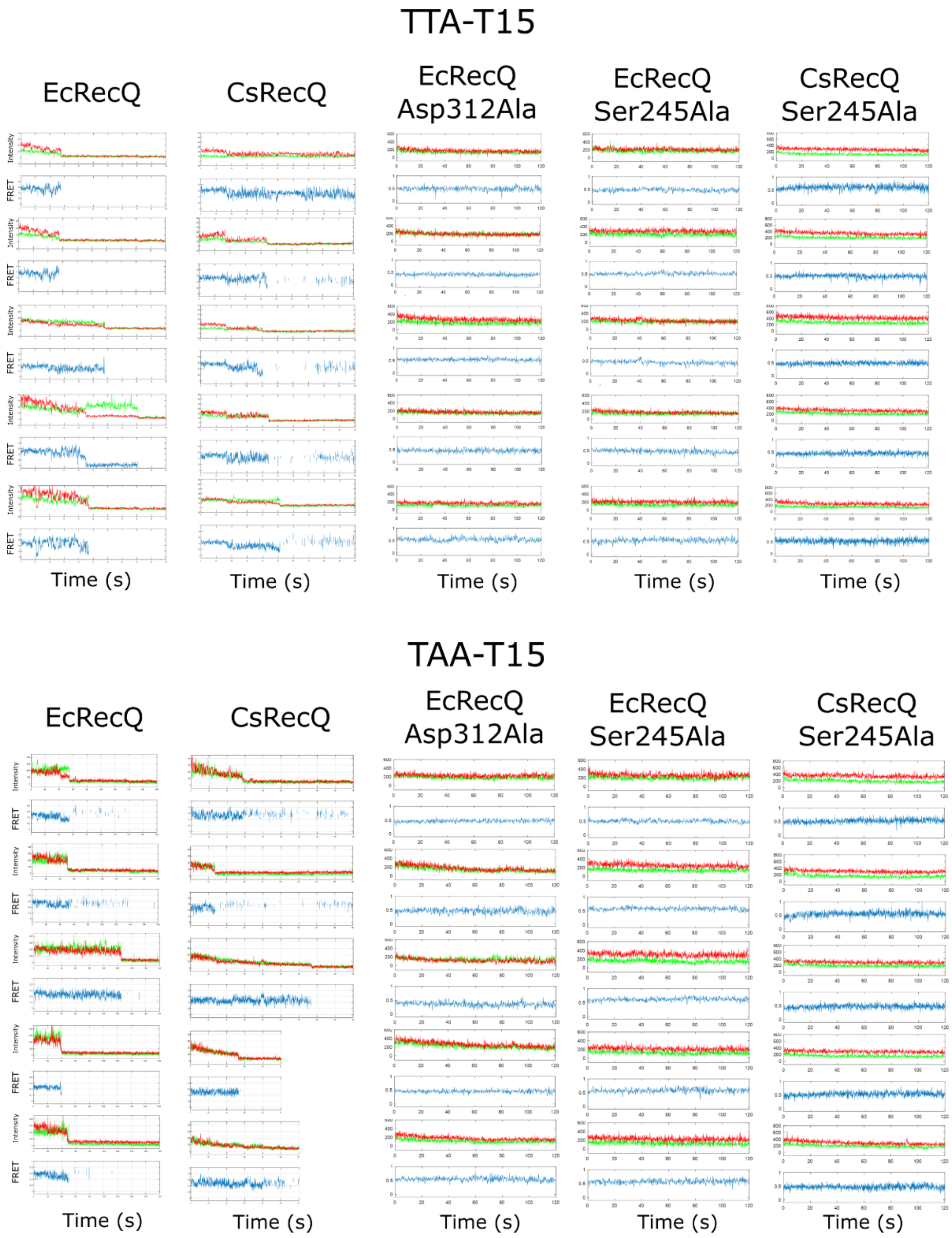

Supplementary Figure 6. Representative single molecule traces of G4 unwinding by RecQ variants.

Supplementary Figure 7

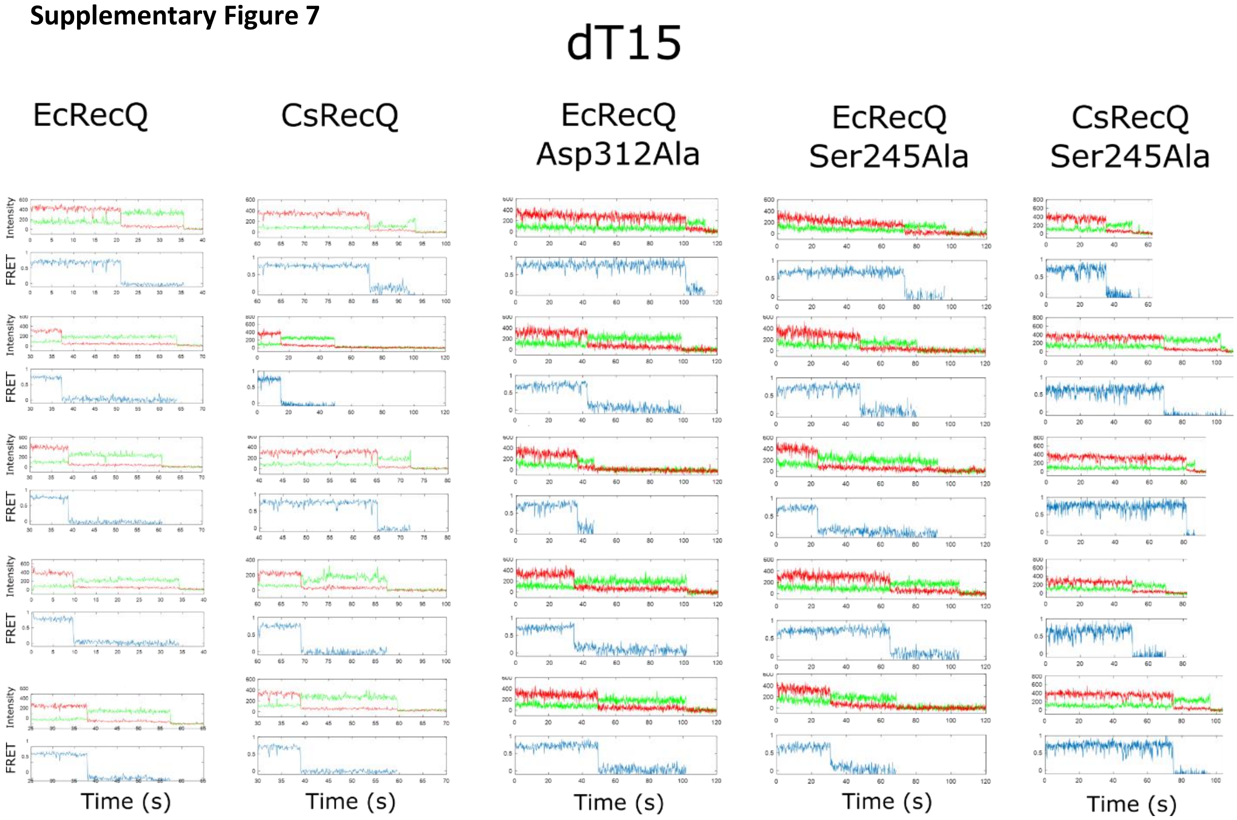

Supplementary Figure 7. Representative single molecule traces of duplex unwinding by RecQ variants.

Supplementary Figure 8.

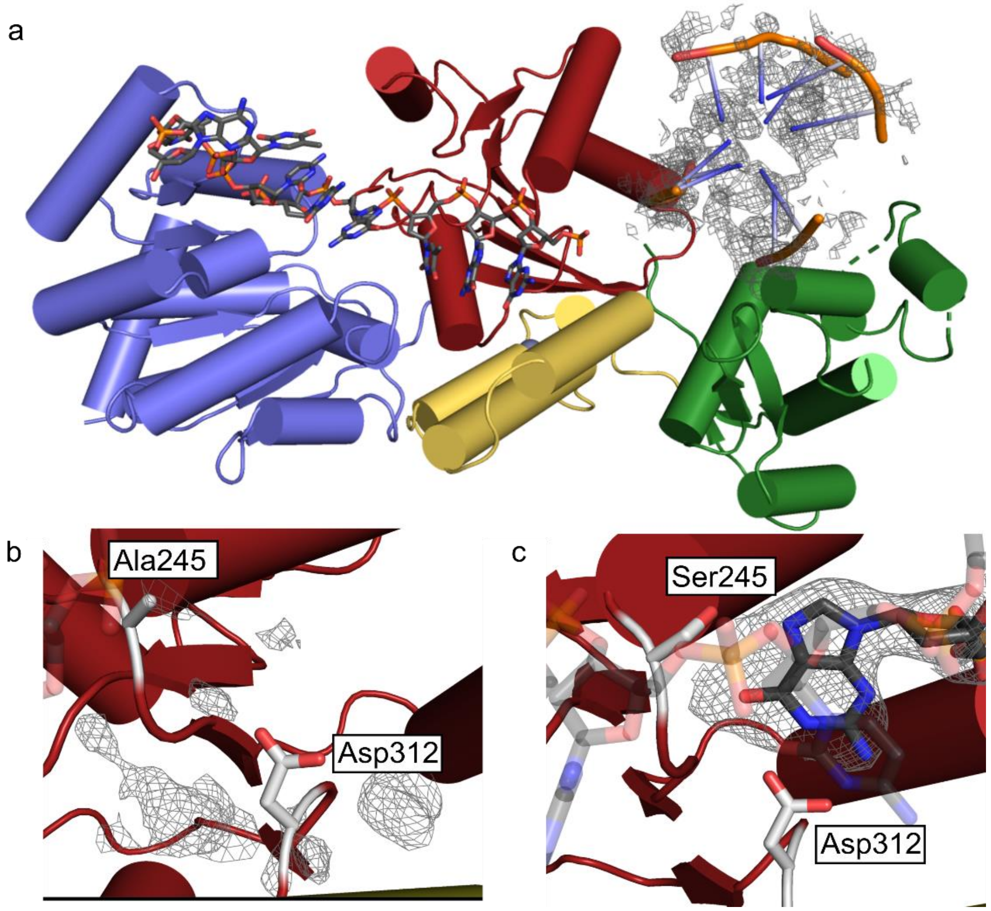

Supplementary Figure 8. Structure of Ser245Ala CsRecQ bound to G4 DNA. a) F_o-F_c electron density map (contoured at 1.7σ) showing significant but discontinuous electron density within the helicase/winged-helix domain cleft. A G4 structure (PDB 143D)[3] is shown within the map for scale. b). F_o-F_c electron density map of the CsRecQ Ser245Ala (contoured at 2.0σ) showing that a guanine is not found in the GSP. c) F_o-F_c omit electron density map of the CsRecQ/G4 product complex (contoured at 2.0σ) with a flipped guanine for comparison.

Supplementary Figure 9

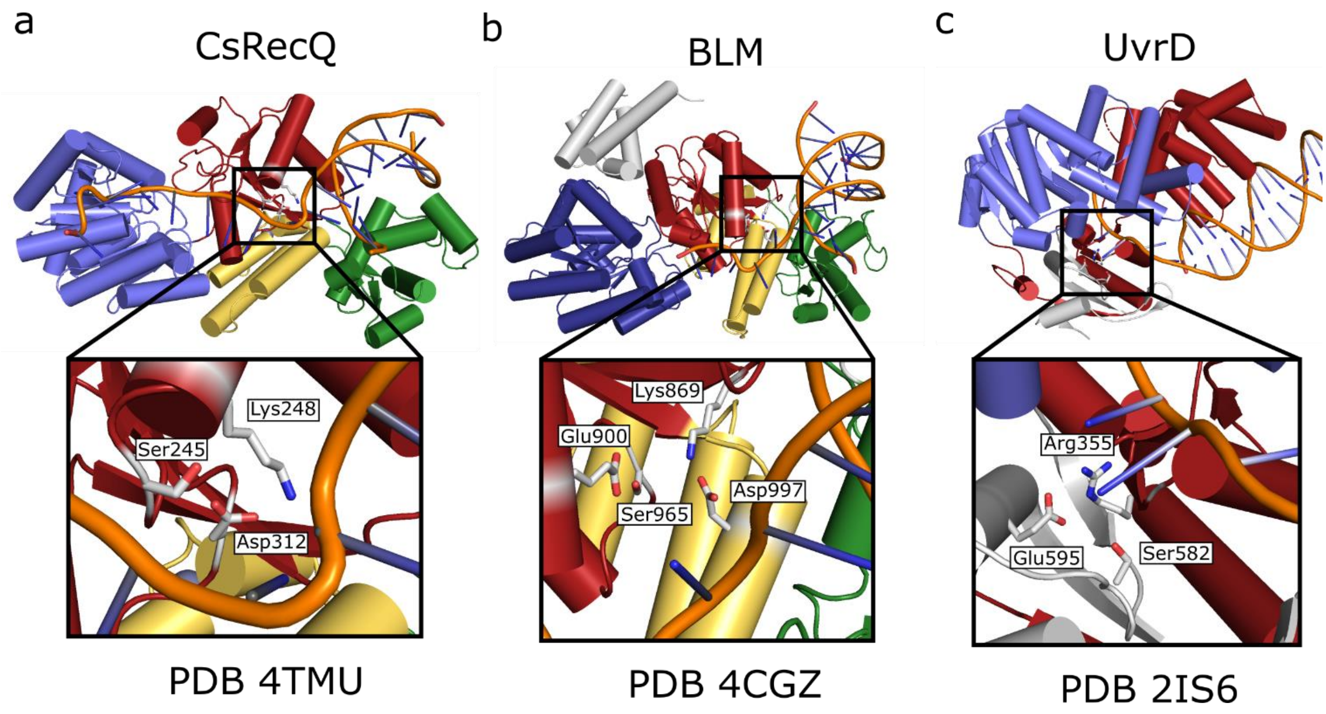

Supplementary Figure 9. Possible structural conservation of the GSP in other G4 resolving helicases. a) The CsRecQ GSP (PDB 4TMU)[1]. b) Pockets with similarity to the RecQ GSP are found in comparable positions in BLM (PDB 4CGZ)[4] and c) in UvrD (PDB 2IS6)[5] helicases.

Supplementary Figure 10

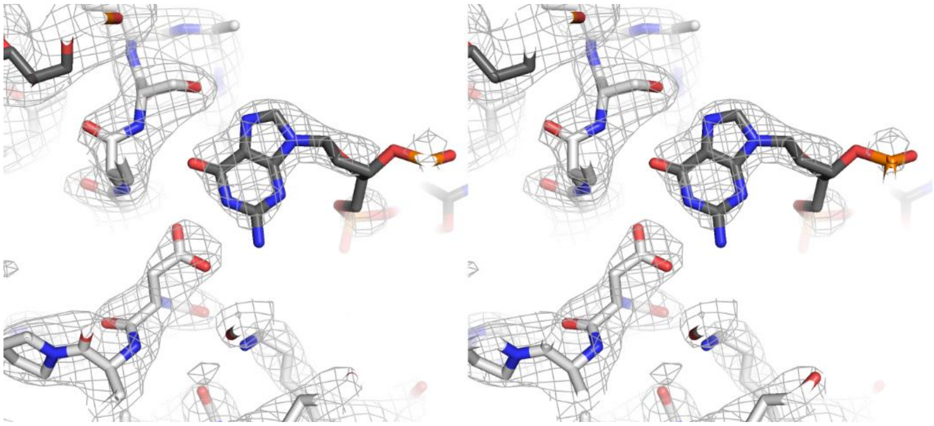

Supplementary Figure 10. Electron density of the refined structure. Stereo image of the refined GSP of the RecQ (white stick) and resolved G4 (grey stick) complex. The $2F_o$-F_c electron density contoured at 1.5σ is shown.

Supplementary Table 1. Nucleotide sequence of the primers used in this study.

**Primer**	**Nucleotide sequence**
G4 DNA	5′-TTA GGG TTA GGG TTA GGG TTA GGG TCG GTG CCT TAC T-3′
F-G4	5′-TTA GGG TTA GGG TTA GGG TTA GGG TCG GTG CCT TAC T-FAM-3′
Duplex 1	5′-GCG TGG GTA ATT GTG CTT CAA TGG ACT GAC-FAM3′
Duplex 2	5′-AAG CAC AAT TAC CCA CGC-3′
Common 18mer	5′-Cy5-GCC TCG CTG CCG TCG CCA-biotin-3′
Non-G4 DNA, T18	5′-TGG CGA CGG CAG CGA GGC-$(T)_{18}$-Cy3-3
TTA-T15 DNA:	5′-TGG CGA CGG CAG CGA GGC TTA GGG TTA GGG TTA GGG TTA GGG-$(T)_{15}$-Cy3-3′
TAA-T15 DNA	5′-TGG CGA CGG CAG CGA GGC TTG GGT AAG GGT AAG GGT AAG GG-$(T)_{15}$-Cy3-3′
c-myc-T15 DNA	5′-TGG CGA CGG CAG CGA GGC GGG TGG GTA GGG TGG G-$(T)_{15}$-Cy3-3′

Supplementary References.

1. Tippana, R., Hwang, H., Opresko, P. L., Bohr, V. A. & Myong, S. Single-molecule imaging reveals a common mechanism shared by G-quadruplex-resolving helicases. *Proc. Natl. Acad. Sci.* **113**, 8448-8453 (2016).
2. Bernstein, D. A., Zittel, M. C. & Keck, J. L. High-resolution structure of the E.coli RecQ helicase catalytic core. *EMBO J.* **22**, 4910-4921 (2003).
3. Wang, Y. and Patel, D. J. Solution structure of the human telomeric repeat d[AG3(T2AG3)3] G-tetraplex. *Structure* **1**(4): 263-282 (1993).
4. Newman, J. A. *et al.* Crystal structure of the Bloom's syndrome helicase indicates a role for the HRDC domain in conformational changes. *Nucleic Acids Res* **43**, 5221-5235 (2015).
5. Lee, J. Y. & Yang, W. UvrD helicase unwinds DNA one base pair at a time by a two-part power stroke. *Cell* **127**, 1349-1360 (2006).
